# Gender- and Age-Specific Differences in the Association of Hyperuricemia and Hypertension: A Cross-Sectional Study

**DOI:** 10.1155/2019/7545137

**Published:** 2019-02-28

**Authors:** Xiaoyun Lin, Xiqian Wang, Xin Li, Lili Song, Zhaowei Meng, Qing Yang, Wenjuan Zhang, Yuxia Gao, Zhenwen Yang, Heng Cai, Bo Bian, Yongle Li, Xuefang Yu, Xin Du, Shaopeng Xu, Jing Nie, Ming Liu, Jinhong Sun, Qing Zhang, Ying Gao, Kun Song, Xing Wang, Li Zhao, Yaguang Fan

**Affiliations:** ^1^Department of Nuclear Medicine, Tianjin Medical University General Hospital, Tianjin, China; ^2^Department of Nephrology, Tianjin Medical University General Hospital, Tianjin, China; ^3^College of Traditional Chinese Medicine, Tianjin University of Traditional Chinese Medicine, Tianjin, China; ^4^Department of Cardiology, Tianjin Medical University General Hospital, Tianjin, China; ^5^Department of Endocrinology and Metabolism, Tianjin Medical University General Hospital, Tianjin, China; ^6^Department of Health Management, Tianjin Medical University General Hospital, Tianjin, China; ^7^Department of Biochemistry and Molecular Biology, School of Basic Medical Sciences, Tianjin Medical University, Tianjin, China; ^8^Tianjin Key Laboratory of Lung Cancer Metastasis and Tumor Microenvironment, Tianjin Lung Cancer Institute, Tianjin Medical University General Hospital, Tianjin, China

## Abstract

**Objective:**

Both hyperuricemia and hypertension have important clinical implications, but their relationship in terms of gender and age is still a matter of debate. In this study, we aimed to explore gender- and age-specific differences in this association between hyperuricemia and hypertension in a Chinese population.

**Methods:**

A total of 78596 ostensibly healthy subjects (47781 men and 30815 women) were recruited. The association between hyperuricemia and hypertension was analyzed by multivariate logistic regression, and the analyses were stratified by gender and age.

**Results:**

Overall prevalence of hypertension and hyperuricemia was significantly higher in males than in females. Increasing trends of hypertension prevalence in both genders as well as hyperuricemia prevalence in females were found along with aging. However, males showed a reduced trend in hyperuricemia prevalence with aging. Higher hypertension and hyperuricemia prevalence was found in young and middle-aged men than in women, but not in elderly people older than 70 years. Significantly increased risk of hypertension from hyperuricemia was found only in men with an adjusted odds ratio of 1.131 (*P* < 0.01), especially in the middle-aged male participants. However, such significant results were not found in women. Similarly, hyperuricemia was also an independent risk factor of increased systolic blood pressure and diastolic blood pressure in males, but not in females.

**Conclusion:**

We observed significantly higher overall prevalence of hyperuricemia and hypertension in men than in women. Men with hyperuricemia (particularly in middle age) had a significantly increased susceptibility of hypertension, while this significant association was not observed in women.

## 1. Introduction

Hypertension is one of the most common cardiovascular diseases and is also the leading preventable cause of premature death worldwide [1]. The global age-standardized prevalence of hypertension was estimated as 24.1% in men and 20.1% in women in 2015. Alarmingly, the number of adults with raised blood pressure increased from 594 million in 1975 to 1.13 billion in 2015, with the increase largely in low-income and middle-income countries [2].

Uric acid (UA) is generated as part of the normal turnover of nucleic acid. UA circulates in plasma predominately in the form of a monovalent sodium salt (urate) [3]. The possibility that UA may be associated with hypertension has been considered for more than a century [4]. However, the interest in the possible link between hypertension and UA waxed and waned during much of the 20th century [5]. It was not until 2001 that a rat model was established to show that the increases in blood pressure were proportional to hyperuricemia induced by providing a uricase inhibitor (oxonic acid) in the diet, which could be ameliorated by UA-lowering drugs [6].

Hyperuricemia has also become a hot topic in recent years [7–9]. However, the opinion on the relationship between hypertension and hyperuricemia is not unanimously accepted as the abovementioned rat model experiment. For instance, several randomized trials have failed to show such a relationship [10, 11]. Palmer et al. [10] argued that body mass index (BMI) could be an important confounder in the development of UA-related conditions rather than UA per se. Some studies also failed to demonstrate that UA-lowering agents could affect endothelial function [11] or the renin-angiotensin system activity [12], both of which were potential mechanisms for the development of hypertension. Another obvious controversy is about the gender- and age-specific differences. Some studies found a more pronounced risk of hypertension associated with hyperuricemia in females [13–15], some in males [16–19], while others in both genders [20, 21]. The age-related variance was even more debated. Lee et al. [22] showed that only people younger than 40 years had significant associations between UA and hypertension, while studies from Yokoi et al. [23] and Cheng et al. [24] demonstrated that only participants older than 40 years had such a relationship. Interestingly, in a Netherland study [25], no age- and sex-related difference was identified in the association between UA and hypertension.

Therefore, to provide more evidence to solve the controversies, the aim of this study is to investigate the association between hyperuricemia and hypertension in a large Chinese population. Also, stratified analyses were conducted to further classify whether this association was gender- and age-specific.

## 2. Methods

### 2.1. Subjects and General Data Collection

This study is a large-scale, single-center, cross-sectional investigation in the Tianjin municipality of China. The database was from Tianjin Medical University General Hospital. A total of 78596 subjects (47781 men and 30815 women) who underwent annual medical examinations at our institution from 2011 to 2015 were enrolled in this study. Information on demographic and lifestyle characteristics, personal medical history, and drug intake information of each subject was collected. The rationale and methodology have been described previously [7–9, 26–34]. All the ostensibly healthy subjects were included in the current analysis. But in order to avoid the influence of confounding factors, exclusion criteria were used for the following situations: participants with past disease histories of hypertension and hyperuricemia; subjects taking any medicine that might influence blood pressure and UA; subjects with endocrinological, hematological, hepatic, renal, gastrointestinal, inflammatory, infectional, oncological, or immunological diseases; and pregnancy.

### 2.2. Ethics, Consent, and Permissions

Informed consent was obtained from all participants, and the Tianjin Medical University General Hospital review board and ethic committee approved this investigation.

### 2.3. Anthropometric Parameters and Blood Pressure

Height, weight, and BMI were measured while the subjects were wearing light clothing and no shoes and in a standing position. BMI was computed as the body weight in kilograms divided by the square of body height in meters. Blood pressure was obtained with the participants in a sitting position after a 5-minute rest using a standard mercury sphygmomanometer. The mean blood pressure was measured three times on the right arm at a minimum interval of 1 minute.

### 2.4. Laboratory Measurements

The concentrations of the following parameters were measured at the central laboratory of Tianjin Medical University General Hospital: UA, alanine aminotransferase (ALT), total bilirubin (TBIL), blood urea nitrogen (BUN), creatinine (Cr), total cholesterol (TC), triglycerides (TG), and fasting glucose (FG). The reference ranges for blood parameters were as follows: ALT 5-40 U/L, TBIL 3.4-20.0 *μ*mol/L, BUN 1.7-8.3 mmol/L, Cr 44-115 *μ*mol/L, TC 3.59-5.18 mmol/L, TG 0.57-1.70 mmol/L, and FG 3.6-5.8 mmol/L.

### 2.5. Definition

Increased systolic blood pressure (SBP) and increased diastolic blood pressure (DBP) were defined as ≥140 mmHg and ≥90 mmHg, respectively. And increased SBP and/or increased DBP was considered as hypertension in line with the US and European guidelines [35, 36]. Criteria for hyperuricemia were determined as >420 *μ*mol/L in men and >360 *μ*mol/L in women [7–9]. Age subgrouping for prevalence analysis was determined according to the following cutoffs: age ≤ 20 years, 20 years < age ≤ 30 years, 30 years < age ≤ 40 years, 40 years < age ≤ 50 years, 50 years < age ≤ 60 years, 60 years < age ≤ 70 years, and age > 70 years. And binary logistic regression was performed according to the following three age categories: young age (≤30 years), middle age (30 years < age ≤ 60 years), and old age (>60 years).

### 2.6. Statistical Analysis

Data were analyzed in males and females separately. Continuous variables including age, BMI, and clinical parameters were expressed as median (interquartile range (IQR)), since they were not consistent in normal distribution, and their differences between subjects with hypertension and those without hypertension were compared with the Mann-Whitney *U* test. Categorical data (prevalence) were expressed as percentages and numbers. The prevalence differences of hypertension and hyperuricemia between males and females were compared with the chi-square test, and the prevalence trend in relation to age group was analyzed with the Cochran-Armitage trend test. The multivariate logistic regression model was used to analyze the association between hyperuricemia and hypertension, and a variable selection procedure was based on the backward LR method. A two-sided *P* value < 0.05 was considered as statistically significant. Statistical assessments were performed using the 3.71 release of SAS Studio for the 9.4M5 version of SAS (SAS Institute Inc., Cary, NC, USA) or IBM SPSS Statistics Version 17.0 software (International Business Machines Corp., Armonk, NY, USA).

## 3. Results

The prevalence of hypertension was significantly higher in males (30.04%) than in females (20.22%) (Table 1). When this comparison was stratified according to age group, the significant difference between males and females was observed in those aged 20-70 years. In addition, hypertension prevalence in both genders increased with age significantly.

The prevalence of hyperuricemia demonstrated a similar pattern of gender difference (Table 2). Also, with the increase in age, the prevalence of hyperuricemia significantly reduced in males while it significantly increased in females.

The basic clinical characteristics of participants with or without hypertension in males and females are summarized in Table 3. The median UA concentrations in male and female hypertensive participants were 365.0 *μ*mol/L and 282.0 *μ*mol/L, respectively, both significantly higher than those in men and women without hypertension. Similarly, age, BMI, and most other clinical parameters were higher in those with hypertension than in those with normotension in both genders, except for levels of TBIL and Cr in men and the TBIL level in women. In addition, the median UA concentrations in males were all higher than those in females whether with hypertension (354.0 *μ*mol/L versus 253.0 *μ*mol/L) or without hypertension (365.0 *μ*mol/L versus 282.0 *μ*mol/L), and the distribution of other parameters in Table 3 was significantly different between males and females.

We further analyzed the relationship between hyperuricemia and hypertension according to gender and age with multivariate logistic regression. And parameters which were significantly different between participants with and without hypertension were adjusted as covariates (Table 4). In males, hyperuricemia was an independent risk factor of hypertension with an adjusted OR of 1.131 (95% CI: 1.073-1.192). When this analysis was repeated according to age, the significant relationship between hyperuricemia and hypertension was only observed in those aged between 30 and 60. On the contrary, no significant relationship between hyperuricemia and hypertension was observed in females. Similarly, hyperuricemia was also an independent risk factor of increased SBP and DBP in males, but not in females, though results of stratified analysis suggested that hyperuricemia increased the risk of increased DBP significantly in females aged over 60. In order to validate our findings, we stratified our data according to the serum Cr level (in accordance with the lower reference range of 44 *μ*mol/L and the upper reference range of 115 *μ*mol/L). We found that the majority of the included cases were within the normal ranges of Cr (male 99.6% and female 98.4%); only 0.4% of men and 0.1% of women had a Cr level higher than 115 *μ*mol/L (Supplementary Table 1). Then, the relationship between hyperuricemia and hypertension was analyzed according to various categories of Cr (Supplementary Tables 2, 3, and 4). We found the same phenomenon as discovered above. Men with hyperuricemia (particularly in middle age) had a significantly increased susceptibility of hypertension, while this significant association was not observed in women. There were too small numbers of cases with the Cr level higher than 115 *μ*mol/L, especially in women, so some analyses could not be performed (Supplementary Tables 1 and 4).

## 4. Discussion

The current study showed that gender and age had a significant impact on the association between hyperuricemia and hypertension in this Chinese population. We found that men with hyperuricemia (particularly in middle age) had a significantly increased susceptibility of hypertension, while no significant association was observed in women.

When exploring the possibility of hyperuricemia as a risk factor of hypertension, it is important to keep in mind the mechanisms of UA production and excretion. Although some mammals, like rats, have uricase (a UA-degrading enzyme), humans lost uricase during the course of evolution. Therefore, as the final metabolite of purine metabolism, UA tends to accumulate in the body, which indeed importantly constitutes the purine skeletons of nucleic acids, adenine, and guanine. There are two routes by which purine enters the human body, through either oral intake or biosynthesis; the latter provides a significantly higher amount of purine than the former. In terms of UA excretion, approximately two thirds occur in the kidney and one third in the digestive tract. Various factors are involved in the processes of UA production and secretion; any malfunction in these steps could lead to hyperuricemia [37].

The biological links between hyperuricemia and hypertension are not fully understood, but continue to be uncovered. There are several lines of evidence for pathomechanistic postulations. A high level of UA can cause arterial stiffness and endothelial dysfunction by two main mechanisms [38, 39]. In the urate crystal mechanism, serum UA itself directly causes arteriosclerosis. Macrophages can engulf the urate crystal, which causes the activation of the Nod-like receptor family protein 3 inflammasome. In the crystal-independent mechanism, the process of UA production by xanthine oxidoreductase is associated with oxidative stress [40], which reduces endothelial nitric oxide bioavailability and stimulates the intracellular renin-angiotensin system. Over time, this process will lead to irreversible vasoconstriction and remodeling in intrarenal vasculature [41]. In addition, the role of insulin resistance can also explain the relationship between UA and hypertension. Elevated insulin levels lead to low urinary ammonium levels and predispose to the precipitation of UA [42]. Insulin resistance acts via distinct and independent mechanisms and contributes to the development of metabolic disorders and hypertension. An *in vivo* and *in vitro* study demonstrated that a high serum UA level could directly induce insulin resistance by inhibiting IRS1 and Akt insulin signaling [43]. In this way, the relationship between hyperuricemia and risks of hypertension in our findings can be comprehended from the molecular explanations.

Hyperuricemia appears to have a stronger impact on the susceptibility to develop hypertension in men than in women, especially in middle-aged males. We try to explain this in the following points. Firstly, sex hormones play a central role in this phenomenon. Circulating UA levels in women are clearly lower than those in men (Table 3), which is putatively the result of the sex hormone in action. Estrogen in women increases renal urate clearance and lowers tubular urate postsecretory reabsorption [44]. Although sex hormone influence will subside in old-aged people after menopause, the serum UA level will be decreased after hormone replacement therapy in postmenopausal women with hyperuricemia [45]. Therefore, the current clinical data (Tables 1 and 2) could putatively reflect that estrogen could decrease the hyperuricemia prevalence in females, which could also suppress the hypertension prevalence in females. Secondly, it has been reported that in middle-aged men in particular, increasing insulin resistance and central obesity is well correlated with a rise in UA [18, 46]. Middle-aged men tend to have more unhealthy lifestyle risk factors that can increase UA, such as more alcohol consumption and less physical exercises [18, 46–48]. On the other hand, middle-aged women are more prone to comply with the health practitioner's advice about keeping fitness, while men tend to be more sedentary [49]. These lifestyle factors will deteriorate the susceptibility in men with hyperuricemia to develop hypertension (Table 4). Thirdly, differences in the expression of urate transporters in nonrenal tissues due to genetic variant SLC2A9 could also regulate blood pressure inhomogeneously in men and women [50]. Indeed, genotype and genetic polymorphism-related changes in the association between UA and blood pressure have also been proven in other laboratories [51, 52].

Interpretation of the present data could be limited by the following factors [1]. This is a cross-sectional, single-center study, which may have selection bias and lack of representativeness, and such a study cannot provide causality information [2]. Some subjects might have white-coat hypertension during the recruitment. Although ambulatory blood pressure monitoring is the best method, it is difficult to be implemented in the setting of an annual medical examination [3]. Serum parameters were measured only once. Sex hormone levels, oxidative stress, insulin resistance, endothelial function, and genotype or genetic polymorphism were not evaluated in the study; thus, their potential confounding effects could not be adjusted for [4]. This is an observational study; interventional studies are needed to clarify whether UA-lowering drugs are useful in preventing development of hypertension, which will validate the findings of the current investigation.

In conclusion, we showed significantly higher overall prevalence of hyperuricemia and hypertension in men than in women, and this difference was more prominent in the young and middle-aged population. Men with hyperuricemia (particularly in middle age) had a significantly increased susceptibility of having hypertension, while this significant association was not observed in women. Further prospective studies in different populations are warranted in investigating the gender- and age-specific association between hyperuricemia and hypertension.

## Figures and Tables

**Table 1:** Prevalence of hypertension on different genders according to age.

Gender	Prevalence (and case number count) in different age subgroups (years)								
	All	Age ≤ 20	20 < age ≤ 30	30 < age ≤ 40	40 < age ≤ 50	50 < age ≤ 60	60 < age ≤ 70	Age > 70	*P* trend
Male									
Normotension	69.96% (33429)	98.21% (55)	89.87% (4651)	82.02% (8623)	69.45% (10173)	61.53% (7160)	53.09% (2158)	36.08% (609)	<0.001
Hypertension	30.04% (14352)	1.79% (1)	10.13% (524)	17.98% (1890)	30.55% (4475)	38.47% (4476)	46.91% (1907)	63.92% (1079)	
Female									
Normotension	79.78% (24583)	100.00% (25)	98.24% (3512)	96.14% (5924)	87.84% (7187)	71.39% (5625)	50.52% (1763)	36.42% (547)	<0.001
Hypertension	20.22% (6232)	0.00% (0)	1.76% (63)	3.86% (238)	12.16% (995)	28.61% (2254)	49.48% (1727)	63.58% (955)	
*P* value	<0.001	0.501	<0.001	<0.001	<0.001	<0.001	0.026	0.842	

**Table 2:** Prevalence of hyperuricemia on different genders according to age.

Gender	Prevalence (and case number count) in different age subgroups (years)								
	All	Age ≤ 20	20 < age ≤ 30	30 < age ≤ 40	40 < age ≤ 50	50 < age ≤ 60	60 < age ≤ 70	Age > 70	*P* trend
Male									
Normotension	79.90% (38177)	52.50% (35)	78.38% (4056)	77.05% (8100)	78.86% (11552)	82.37% (9584)	85.76% (3486)	80.81% (1364)	<0.001
Hypertension	20.10% (9604)	37.50% (21)	21.62% (1119)	22.95% (2413)	21.14% (3096)	17.63% (2052)	14.24% (579)	19.19% (324)	
Female									
Normotension	93.68% (28867)	100.00% (25)	96.03% (3433)	97.01% (5978)	96.36% (7884)	91.83% (7235)	87.85% (3066)	82.96% (1246)	<0.001
Hypertension	6.32% (1948)	0.00% (0)	3.97% (142)	2.99% (184)	3.64% (298)	8.17% (644)	12.15% (424)	17.04% (256)	
*P* value	<0.001	<0.001	<0.001	<0.001	<0.001	<0.001	0.007	0.116	

**Table 3:** Comparisons of medians (IQR) of clinical parameters based on different genders.

Parameters	Male			Female		
	Normotension	Hypertension	*P* value	Normotension	Hypertension	*P* value
Age (years)	44.0 (17.0)	51.0 (15.0)	<0.001	44.00 (18.0)	58.0 (15.0)	<0.001
BMI (kg/m^2^)	25.3 (3.9)	26.8 (4.0)	<0.001	23.1 (4.2)	26.0 (4.7)	<0.001
UA (*μ*mol/L)	354.0 (93.0)	365.0 (99.0)	<0.001	253.0 (72.0)	282.0 (85.0)	<0.001
ALT (U/L)	22.0 (14.0)	24.0 (17.0)	<0.001	15.0 (8.0)	18.0 (11.0)	<0.001
TBIL (*μ*mol/L)	12.8 (6.7)	12.8 (6.6)	0.742	10.4 (6.5)	10.4 (5.1)	0.107
BUN (mmol/L)	4.8 (1.6)	5.0 (1.7)	<0.001	4.2 (1.5)	4.7 (1.6)	<0.001
Cr (*μ*mol/L)	79.0 (14.0)	79.0 (15.0)	0.162	59.0 (12.0)	60.0 (13.0)	<0.001
TC (mmol/L)	5.0 (1.2)	5.22 (1.2)	<0.001	5.0 (1.3)	5.59 (1.3)	<0.001
TG (mmol/L)	1.4 (1.0)	1.64 (1.2)	<0.001	0.97 (0.7)	1.44 (1.0)	<0.001
FG (mmol/L)	5.0 (0.7)	5.3 (0.9)	<0.001	4.8 (0.8)	5.2 (0.9)	<0.001

IQR: interquartile range; BMI: body mass index; UA: uric acid; ALT: alanine aminotransferase; TBIL: total bilirubin; BUN: blood urea nitrogen; Cr: creatinine; TC: total cholesterol; TG: triglycerides; FG: fasting glucose.

**Table 4:** High blood pressure associated with hyperuricemia in different age categories of men and women.

	Male		Female	
	Crude OR (95% CI)	Adjusted OR (95% CI)^#^	Crude OR (95% CI)	Adjusted OR (95% CI)^##^
Hypertension				
All participants	1.387 (1.323-1.454)^∗∗^	1.131 (1.073-1.192)^∗∗^	3.242 (2.662-3.949)^∗∗^	0.797 (0.606-1.050)
Age ≤ 30 (years)	2.036 (1.677-2.471)^∗∗^	1.214 (0.980-1.502)	8.792 (2.544-30.38)^∗∗^	2.827 (0.739-10.810)
30 < age ≤ 60 (years)	1.485 (1.408-1.566)^∗∗^	1.121 (1.058-1.188)^∗∗^	2.761 (2.066-3.689)^∗∗^	0.804 (0.554-1.168)
Age > 60 (years)	1.238 (1.073-1.428)^∗∗^	0.935 (0.772-1.132)	1.397 (1.026-1.901)^∗∗^	0.948 (0.684-1.314)
Increased SBP				
All participants	1.257 (1.190-1.327)^∗∗^	1.085 (1.021-1.153)^∗∗^	3.472 (2.845-4.237)^∗∗^	0.803 (0.605-1.065)
Age ≤ 30 (years)	2.304 (1.823-2.912)^∗∗^	1.355 (1.051-1.747)^∗^	5.561 (0.725-42.64)	1.208 (0.145-10.066)
30 < age ≤ 60 (years)	1.359 (1.275-1.449)^∗∗^	1.044 (0.975-1.119)	3.061 (2.268-4.131)^∗∗^	0.867 (0.588-1.179)
Age > 60 (years)	1.242 (1.078-1.432)^∗∗^	0.930 (0.769-1.125)	1.143 (1.062-1.960)^∗^	0.953 (0.688-1.320)
Increased DBP				
All participants	1.453 (1.383-1.526)^∗∗^	1.102 (1.030-1.179)^∗∗^	2.568 (2.037-3.237)^∗∗^	1.119 (0.877-1.427)
Age ≤ 30 (years)	1.974 (1.594-2.446)^∗∗^	1.141 (0.905-1.438)	7.153 (1.630-31.38)^∗∗^	1.931 (0.233-16. 003)
30 < age ≤ 60 (years)	1.527 (1.446-1.613)^∗∗^	1.152 (1.086-1. 222)^∗∗^	2.233 (1.592-3.132)^∗∗^	0.912 (0.638-1.303)
Age > 60 (years)	1.119 (0.962-1.303)	0.947 (0.808-1.110)	1.746 (1.251-2.437)^∗∗^	1.519 (1.077-2.143)^∗^

OR: odds ratio; CI: confidence interval; SBP: systolic blood pressure; DBP: diastolic blood pressure. ^#^Adjusted for age, BMI, ALT, BUN, TC, TG, and FG. ^##^Adjusted for age, BMI, ALT, BUN, Cr, TC, TG, and FG. ^∗^ *P* < 0.05 and ^∗∗^ *P* < 0.01.

## Data Availability

The data used to support the findings of this study are available from the corresponding authors upon request.

## Supplementary Materials

Supplementary Materials The supplementary materials are provided in order to validate our findings, according to reviewers' suggestions. We calculate our data according to the serum creatinine level (lower reference range 44 *μ*mol/L and upper reference range 115 *μ*mol/L). The relationship between hyperuricemia and hypertension is analyzed according to various categories of creatinine. We validate the same phenomenon that men with hyperuricemia (particularly in middle age) have a significantly increased susceptibility of hypertension, while this significant association is not observed in women.
