# Supplementary material for: Gender- and Age-Specific Differences in the Association of Hyperuricemia and Hypertension: A Cross-Sectional Study
Source: Int J Endocrinol. 2019 Feb 28;2019:7545137. doi: 10.1155/2019/7545137 (PMC6421730; doi:10.1155/2019/7545137)
Supplement: Supplementary Materials — The supplementary materials are provided in order to validate our findings, according to reviewers' suggestions. We calculate our data according to the serum creatinine level (lower reference range 44 μmol/L and upper reference range 115 μmol/L). The relationship between hyperuricemia and hypertension is analyzed according to various categories of creatinine. We validate the same phenomenon that men with hyperuricemia (particularly in middle age) have a significantly increased susceptibility of hypertension, while this significant association is not observed in women. [file 7545137.f1.doc]

| **Supplementary Table 1 Hypertension case number according to gender and Cr level** | | | | | | | | |
| --- | --- | --- | --- | --- | --- | --- | --- | --- |
| Gender | Creatinine level μmol/L | Case Number(%) | Hypertension | | Systolic blood pressure | | Diastolic blood pressure | |
|  |  |  | No(%) | Yes(%) | Normal(%) | High(%) | Normal(%) | High(%) |
| Male | Normal(44-115) | 47579(99.6) | 33316(99.7) | 14263(99.4) | 38482(99.7) | 9097(99.2tal 000000000000000000000000000000000000000000000000000000000000000000000000000000000000000000000000000000000000000000000000) | 35533(99.6) | 12046(99.5) |
|  | Low(<44) | 33(0.1) | 23(0.1) | 10(0.1) | 26(0.1) | 7(0.1) | 23(0.1) | 10(0.1) |
|  | high(>115) | 169(0.4) | 90(0.3) | 79(0.6) | 104(0.3) | 65(0.7) | 114(0.3) | 55(0.5) |
|  | Total | 47781(100) | 33429(100) | 14352(100) | 38612 | 9169(100) | 35670(100) | 12111(100) |
| Female | Normal(44-115) | 30326(98.4) | 24185(98.4) | 6141(98.5) | 25043(98.4) | 5283(98.6) | 27021(98.4) | 3305(98.7) |
|  | Low(<44) | 472(1.5) | 394(1.6) | 78(1.3) | 409(1.6) | 63(0.2) | 432(1.6) | 40(1.2) |
|  | high(>115) | 17(0.1) | 4(0) | 13(0.2) | 5(0) | 12(100) | 12(0.0) | 5(0) |
|  | Total | 30815(100) | 24583 | 6232(100) | 25457(100) | 5358(100) | 27465(100) | 3350(100) |

| **Supplementary Table 2 High blood pressure associated with hyperuricemia in normal Cr level (44-115 μmol/L)** | | | | |
| --- | --- | --- | --- | --- |
|  | Males | | Females | |
|  | Crude OR (95%CI) | Adjusted OR (95%CI)^#^ | Crude OR (95%CI) | Adjusted OR (95%CI)^##^ |
| Hypertension |  |  |  |  |
| All participants | 1.375(1.311-1.442)** | 1.100(1.044-1.159)** | 3.218(2.636-3.930)** | 0.954(0.757-1.203) |
| Age ≤ 30 (years) | 2.044(1.683-2.482)** | 1.172(0.950-1.447) | 9.061(2.611-31.44)** | 3.033(0.787-11.692) |
| 30<age≤60 (years) | 1.477(1.400-1.559)** | 1.088(1.027-1.152)** | 2.742(2.048-3.670)** | 1.000(0.729-1.372) |
| Age>60 (years) | 1.203(1.040-1.391)** | 1.096(0.944-1.272) | 1.409(1.029-1.930)** | 1.010(0.729-1.399) |
| Increased SBP |  |  |  |  |
| All participants | 1.243(1.176-1.313)** | 1.059(0.997-1.124) | 3.433(2.806-4.200)** | 0.974(0.768-1.236) |
| Age ≤ 30 (years) | 2.311(1.827-2.923)** | 1.291(1.004-1.660)* | 5.704(0.742-43.831) | 1.268(0.151-10.624) |
| 30<age≤60 (years) | 1.353(1.269-1.443)** | 1.006(0.940-1.077) | 3.028(2.239-4.095)** | 1.065(0.767-1.479) |
| Age>60 (years) | 1.203(1.041-1.390)** | 1.077(0.928-1.249) | 1.454(1.064-1.989)* | 1.040(0.753-1.438) |
| Increased DBP |  |  |  |  |
| All participants | 1.447(1.377-1.520)** | 1.114(1.056-1.174)** | 2.573(2.036-3.252)** | 1.113 (0.870-1.424) |
| Age ≤ 30 (years) | 1.996(1.611-2.473)** | 1.151(0.912-1.451) | 7.353(1.667-32.36)** | 1.805(0.357-9. 142) |
| 30<age≤60 (years) | 1.519(1.438-1.604)** | 1.122(1.059-1. 189)** | 2.189(1.556-3.081)** | 0.888(0.620-1.273) |
| Age>60 (years) | 1.122(0.961-1.309) | 0.991(0.846-1.161) | 1.799(1.284-2.523)** | 1.555(1.097-2.204)* |
| OR = odds ratio, CI = confidence interval, SBP = systolic blood pressure, DBP = diastolic blood pressure.  #Adjusted for age, BMI, ALT, BUN, TC, TG and FG.  # #Adjusted for age, BMI, ALT, BUN, Cr, TC, TG and FG.  * P<0.05, ** P<0.01. | | | | |

| **Supplementary Table 3 High blood pressure associated with hyperuricemia in Cr level lower than 115 μmol/L (upper reference range)** | | | | |
| --- | --- | --- | --- | --- |
|  | Males | | Females | |
|  | Crude OR (95%CI) | Adjusted OR (95%CI)^#^ | Crude OR (95%CI) | Adjusted OR (95%CI)^##^ |
| Hypertension |  |  |  |  |
| All participants | 1.375(1.311-1.442)** | 1.100(1.044-1.159)** | 3.218(2.636-3.930)** | 0.954(0.757-1.203) |
| Age ≤ 30 (years) | 2.037(1.667-2.473)** | 1.168(0.946-1.441) | 8.792(2.544-30.38)** | 2.827(0.739-10.810) |
| 30<age≤60 (years) | 1.478(1.401-1.559)** | 1.088(1.027-1.152)** | 2.742(2.048-3.670)** | 1.002(0.730-1.375) |
| Age>60 (years) | 1.203(1.040-1.391)** | 1.096(0.944-1.272) | 1.390(1.016-1.901)* | 0.997(0.721-1.378) |
| Increased SBP |  |  |  |  |
| All participants | 1.242(1.176-1.312)** | 1.058(0.997-1.123) | 3.433(2.806-4.200)** | 0.974(0.768-1.236) |
| Age ≤ 30 (years) | 2.311(1.827-2.923)** | 1.284(0.999-1.650) | 5.561(0.725-46.643) | 1.208(0.145-10.066) |
| 30<age≤60 (years) | 1.353(1.269-1.443)** | 1.006(0.939-1.077) | 3.032(2.242-4.101)** | 1.067(0.769-1.482) |
| Age>60 (years) | 1.203(1.040-1.391)** | 1.097(0.944-1.275) | 1.434(1.050-1.958)* | 0.988(0.714-1.357) |
| Increased DBP |  |  |  |  |
| All participants | 1.447(1.377-1.520)** | 1.113(1.055-1.174)** | 2.573(2.036-3.252)** | 1.113 (0.870-1.424) |
| Age ≤ 30 (years) | 1.988(1.604-2.463)** | 1.146(0.909-1.445) | 7.153(1.630-31.38)** | 1.604(0.318-8.082) |
| 30<age≤60 (years) | 1.519(1.438-1.604)** | 1.122(1.059-1. 189)** | 2.195(1.560-3.089)** | 0.891(0.622-1.277) |
| Age>60 (years) | 1.121(0.961-1.308) | 0.991(0.846-1.160) | 1.799(1.284-2.523)** | 1.555(1.097-2.204)* |
| OR = odds ratio, CI = confidence interval, SBP = systolic blood pressure, DBP = diastolic blood pressure.  #Adjusted for age, BMI, ALT, BUN, TC, TG and FG.  # #Adjusted for age, BMI, ALT, BUN, Cr, TC, TG and FG.  * P<0.05, ** P<0.01. | | | | |

| **Supplementary Table 4 High blood pressure associated with hyperuricemia in Cr level higher than 115 μmol/L (upper reference range)** | | | | |
| --- | --- | --- | --- | --- |
|  | Males | | Females | |
|  | Crude OR (95%CI) | Adjusted OR (95%CI)^#^ | Crude OR (95%CI) | Adjusted OR (95%CI)^##^ |
| Hypertension |  |  |  |  |
| All participants | 2.666(1.423-4.994)* | 2.799(1.397-5.608)* |  |  |
| Age ≤ 30 (years) |  |  |  |  |
| 30<age≤60 (years) |  |  |  |  |
| Age>60 (years) |  |  |  |  |
| Increased SBP |  |  |  |  |
| All participants | 1.972(1.042-3.731) | 2.027(0.989-4.156) |  |  |
| Age ≤ 30 (years) |  |  |  |  |
| 30<age≤60 (years) |  |  |  |  |
| Age>60 (years) |  |  |  |  |
| Increased DBP |  |  |  |  |
| All participants | 2.205(1.126-4.320)* | 2.369(1.182-4.750)* |  |  |
| Age ≤ 30 (years) |  |  |  |  |
| 30<age≤60 (years) |  |  |  |  |
| Age>60 (years) |  |  |  |  |
| OR = odds ratio, CI = confidence interval, SBP = systolic blood pressure, DBP = diastolic blood pressure.  #Adjusted for age, BMI, ALT, BUN, TC, TG and FG.  # #Adjusted for age, BMI, ALT, BUN, Cr, TC, TG and FG.  * P<0.05, ** P<0.01. | | | | |
